# BMX/Etk promotes cell proliferation and tumorigenicity of cervical cancer cells through PI3K/AKT/mTOR and STAT3 pathways

**DOI:** 10.18632/oncotarget.17493

**Published:** 2017-04-27

**Authors:** Yuanyuan Li, Nan Cui, Peng-Sheng Zheng, Wen-Ting Yang

**Affiliations:** ^1^ Department of Reproductive Medicine, First Affiliated Hospital of Xi'an Jiaotong University, Xi'an, People's Republic of China; ^2^ Section of Cancer Stem Cell Research, Key Laboratory of Environment and Genes Related to Diseases, Ministry of Education of The People's Republic of China, Xi'an, People's Republic of China

**Keywords:** BMX, cell proliferation, cervical carcinoma, AKT, STAT3

## Abstract

Bone marrow X-linked kinase (BMX, also known as Etk) has been reported to be involved in cell proliferation, differentiation, apoptosis, migration and invasion in several types of tumors, but its role in cervical carcinoma remains poorly understood. In this study, we showed that BMX expression exhibits a gradually increasing trend from normal cervical tissue to cervical cancer *in situ* and then to invasive cervical cancer tissue. Through BMX-IN-1, a potent and irreversible BMX kinase inhibitor, inhibited the expression of BMX, the cell proliferation was significantly decreased. Knockdown of BMX in HeLa and SiHa cervical cancer cell lines using two different silencing technologies, TALEN and shRNA, inhibited cell growth *in vitro* and suppressed xenograft tumor formation *in vivo*, whereas overexpression of BMX in the cell line C-33A significantly increased cell proliferation. Furthermore, a mechanism study showed that silencing BMX blocked cell cycle transit from G0/G1 to S or G2/M phase, and knockdown of BMX inhibited the expression of p-AKT and p-STAT3. These results suggested that BMX can promote cell proliferation through PI3K/AKT/mTOR and STAT3 signaling pathways in cervical cancer cells.

## INTRODUCTION

Cervical carcinoma is the fourth most common cause of cancer and the fourth leading cause of cancer death among women worldwide. Based on the GLOBOCAN estimates, approximately 527,600 new cases of cervical carcinoma were diagnosed, of which more than 84% occurred in developing countries, and 265,700 deaths were reported in 2012 [1]. More than 90% of cervical cancers have been found to be associated with infection with high-risk types of human papillomavirus (HPV) [2]; however, the molecular mechanisms of initiation and cervical carcinogenesis are still unclear. It has been reported that some oncogenes and transcription factors are correlated with cervical cancer and likely involved in the development and progression of cervical cancer, such as SOX2 [3], KLF4 [4], OCT4 [5] and NANOG [6].

Bone marrow X-linked kinase (BMX, also known as Etk) is an intracellular nonreceptor tyrosine kinase member of the Tec family, which includes five mammalian members: Btk (Bruton's tyrosine kinase) [7, 8], Itk (IL-2 inducible *T*-cell kinase) [9], BMX/Etk [10, 11], Tec (tyrosine kinase expressed in hepatocellular carcinoma) [12] and Txk (tyrosine-protein kinase TXK) [13]. BMX has several critical domains, including a Plekstrin homology (PH) domain, Tec homology (TH) domain, Src homology 3 (SH3) domain, Src homology 2 (SH2) domain and a kinase domain (SH1) [14]. BMX can be activated by several chemokines, vascular endothelial growth factor receptors, tumor necrosis factor receptor 2, epidermal growth factor receptor, ErbB3, and integrins [15, 16]. Through its PH domain, BMX directly binds and activates focal adhesion kinase (FAK), RhoA, and PAK1 [17–19]. Moreover, through its SH domain in response to cytokine stimulation, BMX can also interact with p53, Pim-1 and RUFY1 [20–22]. Previous research has shown that BMX can mediate cell proliferation, survival, apoptosis, migration and invasion in various cancers, such as colorectal cancer, breast cancer, prostate cancer and bladder cancer [23–29]. For instance, BMX significantly promoted cell proliferation in human breast cancer MCF-7 cells. Conversely, knockdown of BMX using short interfering RNA in prostate cancer cells inhibited cell proliferation [16, 30], and expression of a kinase-inactive mutant BMX in MDA-MB435 breast cancer cells also suppressed cell proliferation and tumorigenicity [19].

The phosphatidylinositol 3-kinase (PI3K)/protein kinase B (PKB, AKT)/mammalian target of rapamycin (mTOR) signaling pathway regulates various cellular functions that are also critical for tumorigenesis, cell mobility, cell cycle progression, proliferation and survival and therefore is frequently abnormal in many tumors, including colorectal, ovarian, breast and other tumors [31, 32]. Upon stimuli, PI3K converts phosphatidylinositol 4,5-bisphosphate (PIP2) to phosphatidylinositol 3,4,5-trisphosphate (PIP3), which recruits BMX, AKT and its activating kinase PDK1 through a PH domain. Following AKT activation, mTOR can be activated [33, 34]. Signal transducer and activator of transcription 3 (STAT3) is a transcriptional factor that has been demonstrated to be constitutively activated in several cancers, such as breast cancer, lung, colorectal and prostate cancers. STAT3 mediates the expression of many genes in response to cell stimuli and involved in cell proliferation, apoptosis, migration, survival and tumorigenesis [35, 36]. BMX has previously been verified as an activator of STAT3, and can phosphorylate STAT3 *in vitro* [37, 38].

However, the function of BMX in cervical cancer is still poorly understood. In this study, we aimed to explore the role of BMX during the development and progression of cervical cancer, and we are the first to report that BMX can promote cell proliferation and tumor formation in cervical cancer by activating PI3K/AKT and STAT3 signaling pathways.

## RESULTS

### The expression of BMX in the normal human cervix and cervical cancerous lesions

**Figure 1:**
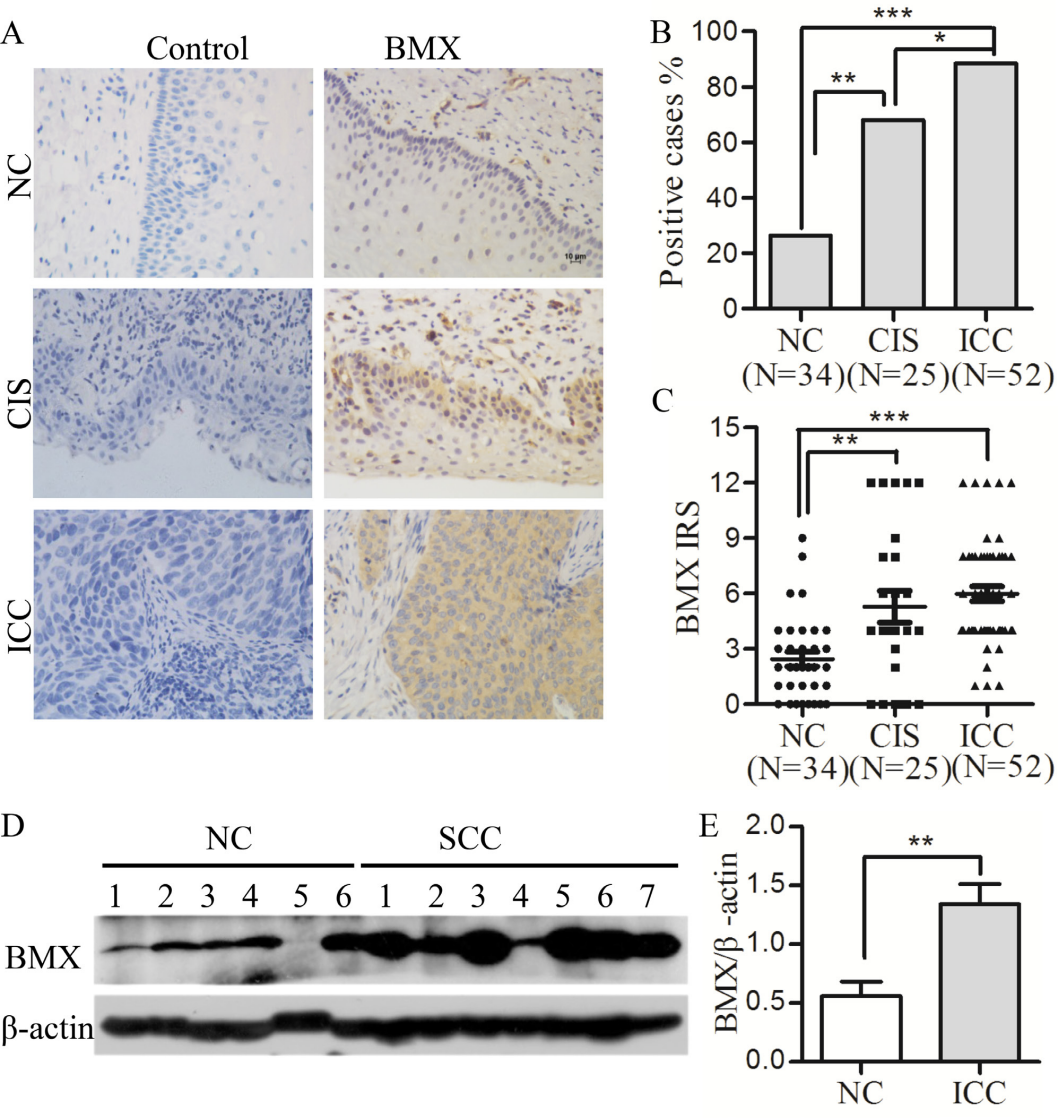
BMX expression is up-regulated in cervical carcinomas (**A**) Immunohistochemistry (IHC) for BMX expression is shown in the normal human cervix (NC, *n* = 34), cervical carcinoma *in situ* (CIS, *n* = 25) and invasive cervical carcinoma (ICC, *n* = 52); scale bar is 10 μm. (**B**) Analysis of the percentage of BMX-positive cells in NC, CIS and ICC using a *chi-square* test. (**C**) The average immunoreactivity score (IRS) of BMX staining in NC, CIS and ICC; *one-way* ANOVA was performed. (**D**) Western blot analysis of BMX expression in normal cervix (NC, *n* = 6) and invasive cervical carcinoma (ICC, *n* = 7) is shown. (**E**) The relative quantitative analysis of BMX expression according to western blot results using Quantity One software; a *t*-test was performed. Values are shown as the mean ± SEM, *p < 0.05, **p < 0.01, and ***p < 0.001.

Although, BMX has been reported in glioblastoma stem cells and various somatic carcinomas, such as prostate cancer, breast cancer and bladder cancer [39, 40], the function of BMX in cervical carcinoma is still not known. To investigate whether BMX is involved in cervical carcinogenesis, the expression of BMX was detected in normal cervix (NC), cervical carcinoma *in situ* (CIS) and invasive cervical carcinoma (ICC) samples using immunohistochemistry (Figure 1A). The percentage of positive BMX staining was significantly increased from 26.47% (NC samples, 9/34) to 68.00% (CIS samples, 17/25) and 88.46% (ICC samples, 46/52, Figure 1B), and the immunoreactivity score (IRS) of BMX staining was also increased from 2.441 ± 2.286 (NC samples) to 5.280 ± 4.326 (CIS samples) and 5.981 ± 2.920 (ICC samples) (Figure 1C), indicating that BMX may be increased during the progression of human cervical carcinoma. Furthermore, a western blot was used to analyze the expression of BMX in 6 normal cervical and 7 cervical cancer tissues, all of which were selected randomly. As shown in Figure 1D, the expression of BMX was significantly higher in cervical carcinoma tissues than in normal cervical tissues (Figure 1E, *p* < 0.01). All of these results indicated that BMX was increased in cervical carcinoma and strongly suggested that BMX must be related to cervical carcinogenesis.

### BMX promoted proliferation of cervical cancer cells *in vitro*

**Figure 2:**
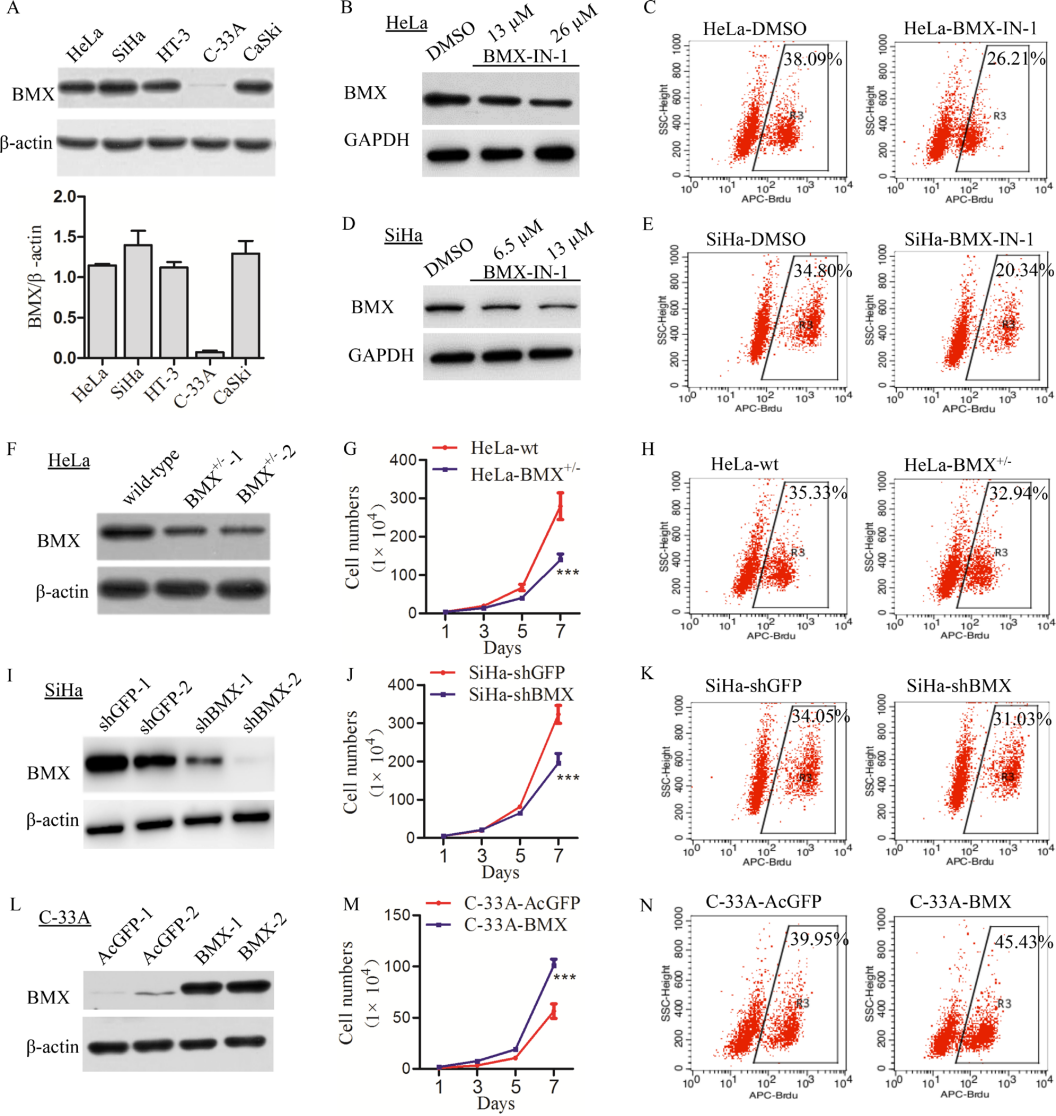
BMX promoted the proliferation of cervical carcinoma cells *in vitro* (**A**) The expression of BMX in cervical cancer cell lines was detected with a western blotting assay, and the quantitative analysis using Quantity One software is shown. (**B**) HeLa cells were treated with DMSO as control, 13 μM and 26 μM BMX-IN-1, and the expression of BMX was determined using a western blotting. (**C**) Treated HeLa cells with DMSO, and 26 μM BMX-IN-1, flow cytometry analysis was used to assess the cell proliferation with Brdu incorporation, *p* < 0.001. (**D**) SiHa cells were treated with DMSO, 6.5 μM and 13 μM BMX-IN-1, and the expression of BMX was determined. (**E**) Treated SiHa cells with DMSO, and 13 μM BMX-IN-1, the cell proliferation with Brdu incorporation was assessed, *p* < 0.001. (**F**) A western blotting assay was used to detect the expression of BMX in TALEN-mediated HeLa BMX-knockdown clones. (**G**) Growth curves and (**H**) flow cytometry analysis (*p* < 0.05) were used to assess the proliferation of HeLa-wt/BMX^+/−^ cells. (**I**) A western blotting assay was used to detect the expression of BMX in shRNA-mediated SiHa BMX-knockdown clones. (**J**) Growth curves and (**K**) flow cytometry analysis (*p* < 0.05) were used to assess the proliferation of SiHa-shGFP/shBMX cells. (**L**) A western blotting assay was used to detect the expression of BMX in C-33A BMX-overexpressing clones (transfected with AcGFP or BMX-overexpression plasmid). (**M**) Growth curves and (**N**) flow cytometry analysis (*p* < 0.05) were used to assess the proliferation and viability of C-33A-AcGFP/BMX-overexpressing cells. Values are shown as the mean ± SEM from three independent experiments (*t*-test, *p < 0.05, **p < 0.01, ***p < 0.001 vs the corresponding control).

Western blotting was used to detect the expression of BMX in cervical cancer cell lines, and a high level of BMX expression was observed in HeLa, SiHa, HT-3 and CaSki cells, and a low level of BMX expression was observed in C-33A cells (Figure 2A). To explore the function of BMX in cervical cancer cells, BMX-IN-1, a potent, selective, and irreversible BMX kinase inhibitor, was used to attenuate the expression of BMX in HeLa and SiHa cells. Western blot results showed that the expression of BMX was decreased in both BMX-IN-1-treated HeLa and SiHa cells (Figure 2B and 2D). Flow cytometry analysis was used to detect the cell proliferation, and the results shown that the percentage of APC-Brdu-positive cells in HeLa-BMX-IN-1 (26.21%) and SiHa-BMX-IN-1 (20.34%) was significantly lower than that in control HeLa-DMSO (38.09%) and SiHa-DMSO (34.80%), respectively (Figure 2C and 2E, *p* < 0.001). Furthermore, cell viability, as determined by an MTT assay, was much lower in BMX-IN-1 treated HeLa and SiHa cells than the control cells (Supplementary Figure 2A and 2D, *p* < 0.001). These results suggested that attenuation of the expression of BMX by BMX-IN-1 treatment attenuated the cell proliferation in HeLa and SiHa cells.

Furthermore, a recombinant BMX-TALEN plasmid was transfected into HeLa cells to knockdown BMX expression (Figure 2F and Supplementary Figure 1). The results of the cell growth curve assay revealed that the cell growth of HeLa-BMX^+/−^ cells was slower than that of the HeLa-wt cells (Figure 2G), and flow cytometry analysis showed that the percentage of APC-Brdu-positive cells in HeLa-BMX^+/−^ (32.94%) was lower than that in HeLa-wt cells (35.33%) (Figure 2H, *p* < 0.05). Moreover, the expression of BMX was also knocked down in SiHa cells using an shBMX plasmid (Figure 2I). Accordingly, the cell growth of SiHa-shBMX cells was also slower than that of the SiHa-shGFP cells (Figure 2J), and flow cytometry analysis showed that the percentage of APC-Brdu-positive cells in SiHa-shBMX (31.03%) was lower than that in SiHa-shGFP cells (34.05%) (Figure 2K, *p* < 0.05). Furthermore, cell viability, as determined by an MTT assay, was much lower in BMX-Knockdown HeLa and SiHa cells than the control cells (Supplementary Figure 2G and 2H). These suggesting that knockdown of BMX expression in cervical cancer cells can attenuate cell proliferation and viability.

Moreover, BMX was stably overexpressed in C-33A cells using a recombinant plasmid, and a western blotting assay was used to detect the expression of BMX in C-33A-AcGFP and C-33A-BMX cells (Figure 2L). The cell growth curves revealed that the cell growth of C-33A-BMX cells was much faster than that of the C-33A-AcGFP cells (Figure 2M), and flow cytometry analysis showed that the percentage of APC-Brdu-positive cells in C-33A-BMX (45.43%) was higher than that in C-33A-AcGFP cells (39.95%) (Figure 2N, *p* < 0.05). Cell viability was also much higher in C-33A-BMX cells than C-33A-AcGFP cells (Supplementary Figure 2I). All of these data indicated that BMX could promote the proliferation of cervical carcinoma cells.

### BMX promoted tumor formation of cervical cancer cells *in vivo*

**Figure 3:**
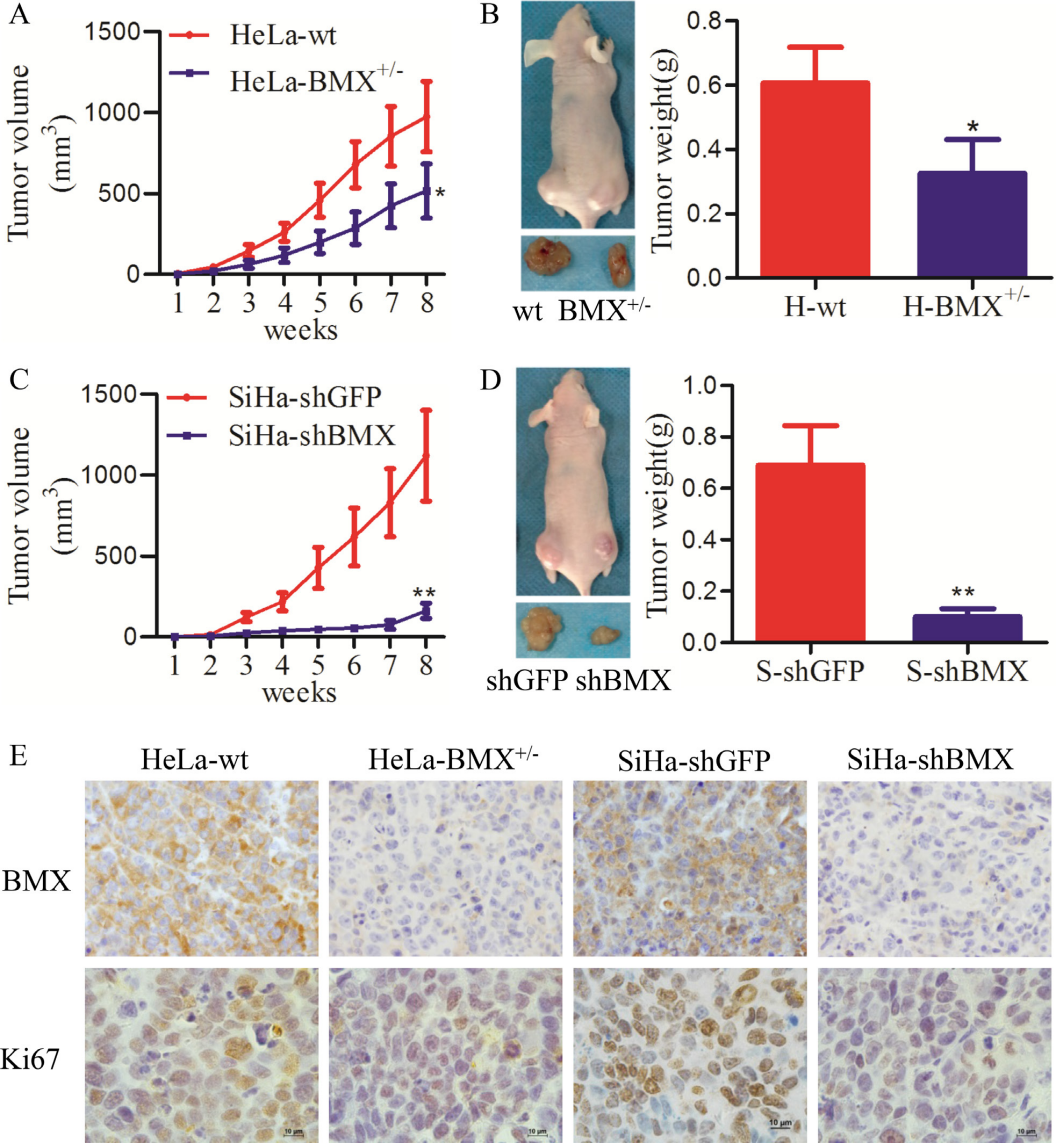
BMX promoted tumor growth of cervical carcinoma *in vivo* (**A**) The tumor growth curves of BALB/c nude mice with HeLa-wt and HeLa-BMX^+/−^ cells (1 × 10^6^) are shown. Tumors were measured weekly. (**B**) Xenografts were dissociated and weighed at 8 weeks post-transplant for HeLa-wt (left) and HeLa-BMX^+/−^ (right) cells. (**C**) The tumor growth curves of BALB/c nude mice with SiHa-shGFP and SiHa-shBMX cells. (**D**) Xenografts were dissociated and weighed at 8 weeks post-transplant for SiHa-shGFP (left) and SiHa-shBMX (right) cells. The data were analyzed and are shown as the mean ± SEM. Tumor growth curves were determined using *two-way* ANOVA, and weights were determined using a *t*-test (*p < 0.05, **p < 0.01). (**E**) Immunochemistry of tumor xenografts stained with BMX and Ki67 is shown. Ki67 is a well-known cell proliferation marker. Scale bar = 10 μm.

To investigate the effect of BMX on tumor formation *in vivo*, 1 × 10^6^ control and BMX-modified cells were implanted into female nude mice. As shown in Figure 3, the volume of tumors that formed from BMX-knockdown cells (HeLa-BMX^+/−^ and SiHa-shBMX) was much less than that of tumors formed from the control cells (HeLa-wt and SiHa-shGFP) (Figure 3A and 3C). The average weight of xenografted tumors that formed from BMX-knockdown cells (HeLa-BMX^+/−^ and SiHa-shBMX) was also less than that of tumors formed from the control cells (HeLa-wt and SiHa-shGFP) (Figure 3B and 3D). However, both the C-33A-AcGFP and C-33A-BMX cells failed to form xenografted tumors in female nude mice. These results suggested that BMX can promote tumor formation of cervical cancer cells *in vivo*.

To determine whether BMX enhanced tumor formation by promoting cell proliferation, immunohistochemistry was used to detect the expression of BMX and Ki67 (a well-known cell proliferation maker) in the xenografted tumors. As shown in Figure 3E, the expression of BMX and Ki67 in the xenografted tumors that formed from HeLa-BMX^+/−^ and SiHa-shBMX cells was much less than that observed in tumors formed from the control cells (HeLa-wt and SiHa-shGFP cells). These data suggested that BMX promoted tumor formation of cervical cancer cells *in vivo*, which must be dependent on the effect of BMX on cell proliferation.

### BMX promoted cell proliferation by accelerating cell cycle transition from G0/G1 to S or G2/M phase

**Figure 4:**
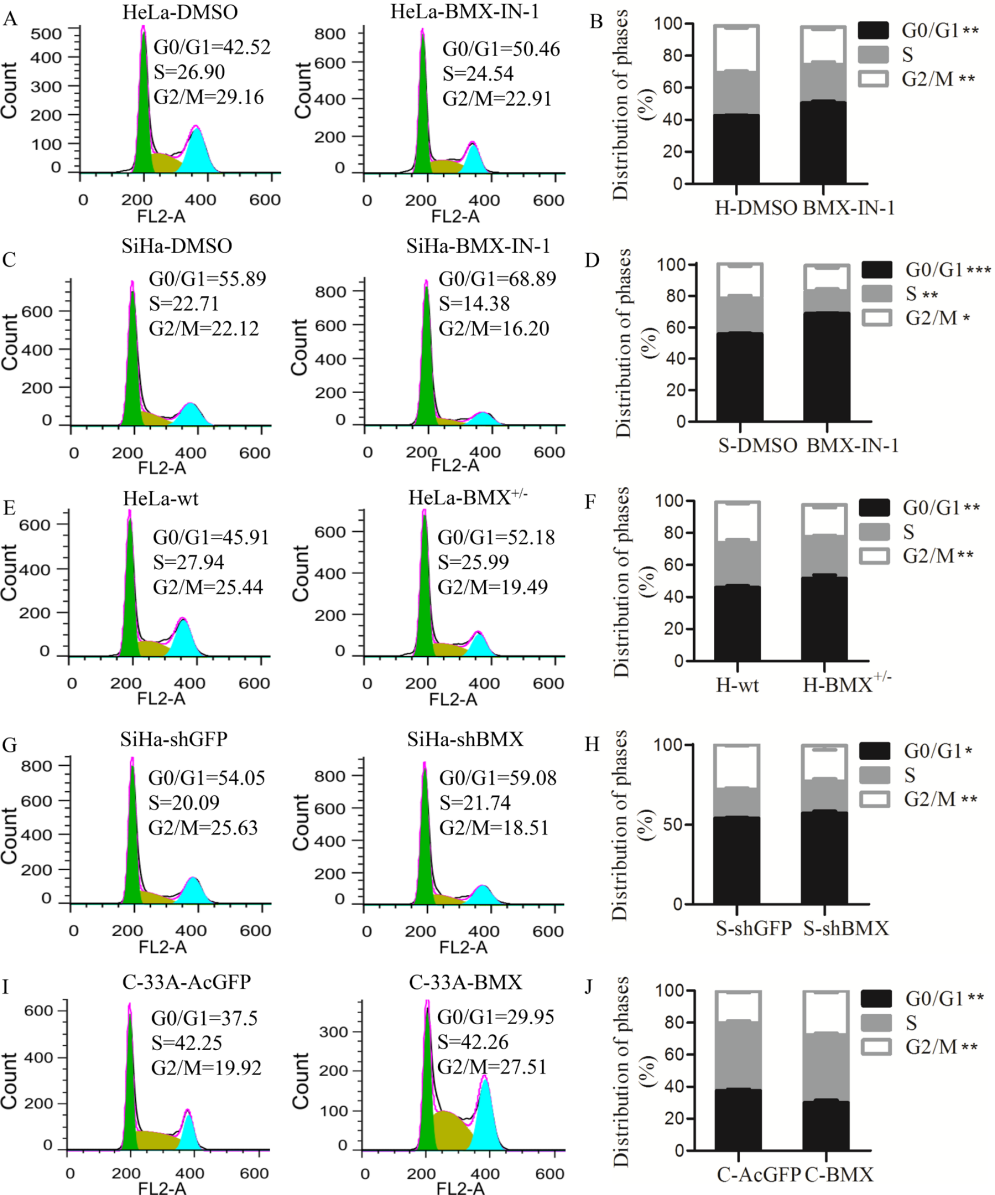
BMX promoted cell proliferation by accelerating cell cycle entry (**A**) Treated HeLa cells with DMSO and 26 μM BMX-IN-1, flow cytometry analysis was used to analyzed the cell cycle distribution. Data were processed using FlowJo 7.6 software. The statistical analysis is shown in (**B**). (**C**) Treated SiHa cells with DMSO and 13 μM BMX-IN-1, and analyzed the cell cycle distribution. The statistical analysis is shown in (**D**). (**E**) Cell cycle data of HeLa-wt and HeLa-BMX^+/−^ cells was processed, and the statistical analysis is shown in (**F**). (**G**) Cell cycle data of SiHa-shGFP and SiHa-shBMX cells was processed, and the statistical analysis is shown in (**H**). (**I**) Cell cycle data of C-33A-AcGFP and C-33A-BMX cells was processed, and the statistical analysis is shown in (**J**). A *t*-test was used for the statistical analysis, *p < 0.05, **p < 0.01.

To investigate how BMX protein promoted cell proliferation, flow cytometry analysis was used to examine the changes of cell cycle in the BMX-modified cells and control cells. Treating HeLa and SiHa cells with BMX-IN-1, the number of cells in G0/G1 phase was much higher in the HeLa-BMX-IN-1 (50.46%) and SiHa-BMX-IN-1 cells (68.89%) than in the HeLa-DMSO (42.52%) and SiHa-DMSO groups (55.89%) (HeLa, Figure 4A and 4B, *p* < 0.01, SiHa, Figure 4C and 4D, *p* < 0.001), while the number of cells in G2/M phase was lower in the HeLa-BMX-IN-1 cells (22.91%) and SiHa-BMX-IN-1 cells (16.20%) than in HeLa-DMSO (29.16%) and SiHa-DMSO groups (22.12%), respectively (HeLa, Figure 4A and 4B, *p* < 0.01, SiHa, Figure 4C and 4D, *p* < 0.05). Furthermore, the proportion of cells in G0/G1 phase was also higher in the HeLa-BMX^+/−^ cells (52.18%) than in the HeLa-wt cells (45.91%, *p* < 0.01), while the proportion of cells in G2/M phase was lower in the HeLa-BMX^+/−^ cells (19.49%) than in the HeLa-wt cells (25.44%, Figure 4E and 4F, *p* < 0.01). Moreover, the proportion of cells in G0/G1 phase was much higher in the SiHa-shBMX cells (59.08%) than in the SiHa-shGFP cells (54.05%, *p* < 0.05), while the proportion of cells in G2/M phase was much lower in the SiHa-shBMX cells (18.51%) than in the SiHa-shGFP cells (25.63%, Figure 4G and 4H, *p* < 0.01). These results suggested that BMX knockdown in HeLa and SiHa cells increased the number of cells in G0/G1 phase and decreased the number of cells in S or G2/M phase. In contrast, the proportion of cells in G0/G1 phase was much lower in the BMX-overexpressing C-33A cells (29.95%) than in the C-33A-AcGFP cells (37.5%, *p* < 0.01), while the proportion of C-33A-BMX cells in G2/M phase (27.51%) was much higher than that of the C-33A-AcGFP cells (19.92%, Figure 4I and 4J, *p* < 0.01), suggesting that BMX overexpression in C-33A cells decreased the number of cells in G0/G1 phase and increased the number of cells in G2/M phase. All of these results suggested that BMX promoted the proliferation of cervical cancer cells by decreasing the number of cells in G0/G1 phase and increasing the number of cells in S or G2/M phase.

### BMX promoted proliferation of cervical cancer cells by activating PI3K/AKT and STAT3 signaling pathways

**Figure 5:**
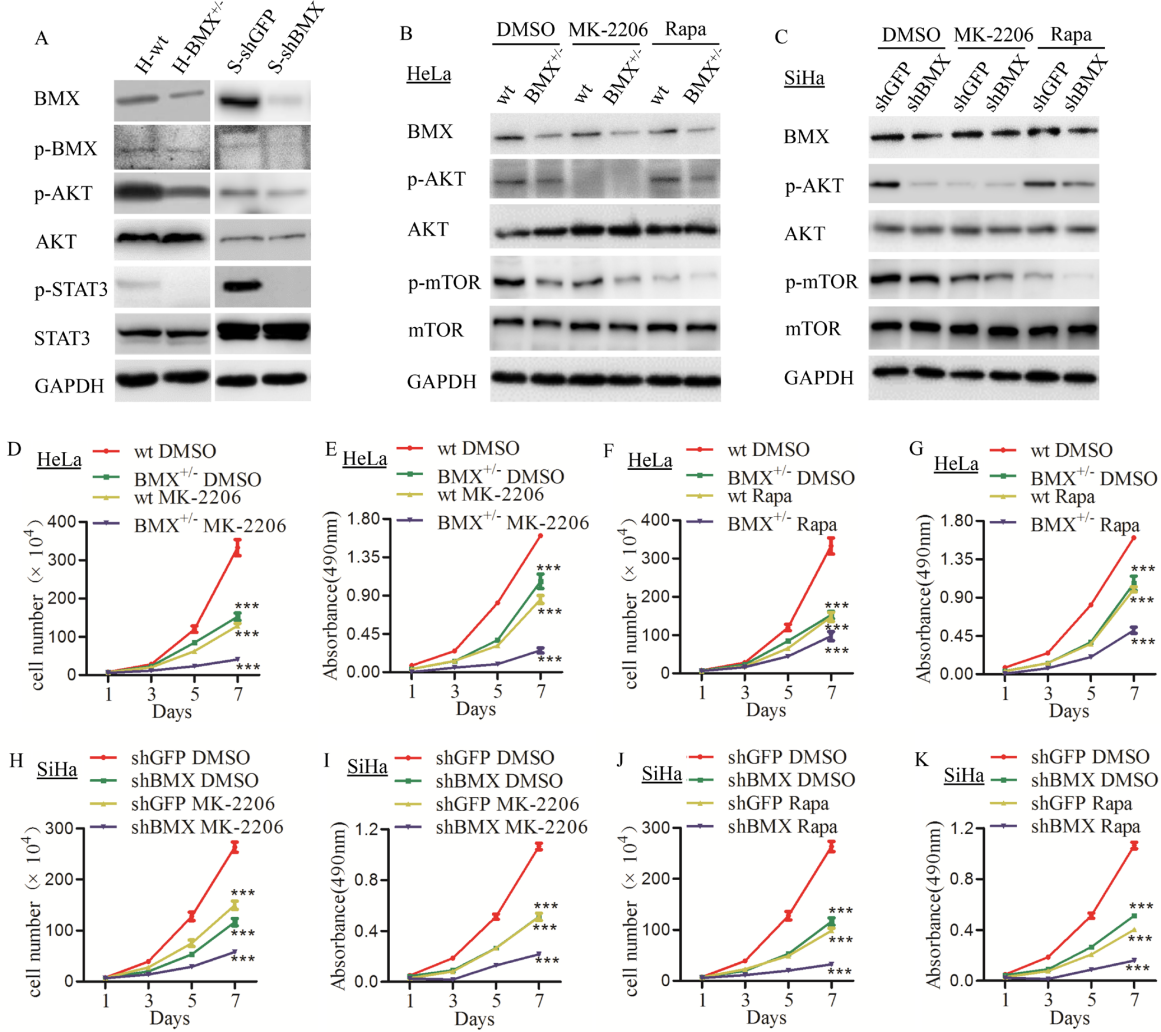
BMX promoted the proliferation of cervical cancer cells through the AKT/mTOR pathway (**A**) Western blot detection of BMX, p-BMX, p-AKT, AKT, p-STAT3 and STAT3 in HeLa-wt/BMX^+/−^ and SiHa-shGFP/shBMX cell lines. (**B**) HeLa-wt/BMX^+/−^ clones were treated with DMSO control, the selective AKT inhibitor MK-2206 (2 μM) and mTOR inhibitor rapamycin (2 μM), and BMX, p-AKT, AKT, p-mTOR and mTOR expression was detected using a western blotting assay. (**C**) SiHa-shGFP/shBMX cells were treated with DMSO control, the selective AKT inhibitor MK-2206 (3 μM) and mTOR inhibitor rapamycin (3 μM), and BMX, p-AKT, AKT, p-mTOR and mTOR expression was detected using a western blotting assay. (**D**) Growth curves and (**E**) MTT assays were performed to assess the proliferation and viability of HeLa-wt DMSO, BMX^+/−^ DMSO, wt MK-2206 and BMX^+/−^ MK-2206 cells. (**F**) The growth curves and (**G**) MTT assay results of HeLa-wt DMSO, BMX^+/−^ DMSO, wt rapamycin and BMX^+/−^ rapamycin cells are shown. (**H**) The growth curves and (**I**) MTT assay results of SiHa-shGFP DMSO, shBMX DMSO, shGFP MK-2206 and shBMX MK-2206 cells are shown. (**J**) The growth curves and (**K**) MTT assay results of SiHa-shGFP DMSO, shBMX DMSO, shGFP rapamycin and shBMX rapamycin cells are shown. The data were compared as follows: BMX-knockdown (HeLa-BMX^+/−^ and SiHa-shBMX) groups vs. control (HeLa-wt and SiHa-shGFP) groups and all groups treated with MK-2206/rapamycin vs. DMSO-treated groups. Growth curve and MTT assay data were analyzed using *two-way* ANOVA, and results are shown as the mean ± SEM. *p < 0.05, **p < 0.01, ***p < 0.001, ^#^*p* > 0.05.

A previous study reported that BMX could be activated by tyrosine phosphorylation downstream of PI3K, and AKT is an essential factor in the PI3K/AKT signaling pathway, which is important for cell proliferation [41–43]. To investigate whether BMX promotes cell proliferation and tumor formation by activating AKT, a western blot was used to detect the protein level of p-AKT and AKT in HeLa-wt/HeLa-BMX^+/−^ and SiHa-shGFP/SiHa-shBMX cells. As shown in Figure 5A and Supplementary Figure 5A, the expression of p-BMX and BMX was much lower in HeLa-BMX^+/−^ and SiHa-shBMX cells than in the control cells (HeLa-wt and SiHa-shGFP cells, respectively), and the expression of p-AKT was also much lower in HeLa-BMX^+/−^ and SiHa-shBMX cells than in the control cells (HeLa-wt and SiHa-shGFP cells, respectively), while the total AKT level was not changed, suggesting that knockdown of BMX can decrease the expression of p-AKT/AKT in HeLa-BMX^+/−^ and SiHa-shBMX cells.

Moreover, BMX has also been identified as an activator of STAT3 in glioblastoma stem cells [38]. Constitutive activation of STAT3 accelerates cell proliferation, migration and tumor formation in several tumors, such as breast cancer and colorectal cancer. As shown in Figure 5A and Supplementary Figure 5A, the expression of p-STAT3 was much lower in both HeLa-BMX^+/−^ and SiHa-shBMX cells than in the control cells (HeLa-wt and SiHa-shGFP cells, respectively), while the total STAT3 level was not changed, suggesting that knockdown of BMX could also decrease the expression of p-STAT3/STAT3 in HeLa-BMX^+/−^ and SiHa-shBMX cells. All of these results indicated that BMX could enhance the activity of p-AKT/AKT and p-STAT3/STAT3.

To further confirm that BMX promotes cell proliferation and tumor formation through the AKT/mTOR pathway in cervical cancer cells, the AKT inhibitor MK-2206 and mTOR inhibitor rapamycin were used in HeLa-wt, HeLa-BMX^+/−^, SiHa-shGFP and SiHa-shBMX cell lines. As shown in Figure 5B and Supplementary Figure 5B, in DMSO-treated HeLa-wt/BMX^+/−^ cells, the expression of both p-AKT and p-mTOR was lower in HeLa-BMX^+/−^ cells than in HeLa-wt cells; in MK-2206-treated HeLa-wt/BMX^+/−^ cells, the expression of both p-AKT and p-mTOR were inhibited compared with the DMSO-treated control group; in rapamycin-treated HeLa-wt/BMX^+/−^ cells, the expression of p-mTOR but not p-AKT was inhibited compared with the DMSO-treated control group. This result verified that BMX can activate the phosphorylation of AKT, and mTOR, as a canonical downstream factor, was also influenced. The results of cell proliferation assays are shown in Figure 5D and 5E, of the DMSO-treated cells, the HeLa-BMX^+/−^ cells grew much more slowly than the HeLa-wt cells; of the MK-2206-treated cells, the growth of the HeLa-wt and HeLa-BMX^+/−^ clones was more inhibited than the growth of the DMSO-treated control cells, respectively. As shown in Figure 5F and 5G, the growth of rapamycin-treated HeLa-wt and HeLa-BMX^+/−^ cells was also more inhibited than that of the DMSO-treated control cells, respectively. In SiHa-shGFP/shBMX cells, we obtained the same results with a western blotting assay (Figure 5C) and with the quantitative analysis using Quantity One software (Supplementary Figure 5C). The cell proliferation of MK-2206- (Figure 5H and 5I) and rapamycin (Figure 5J and 5K)-treated SiHa-shGFP and SiHa-shBMX cells was more inhibited than that of the DMSO-treated control cells, respectively. These results suggested that suppression of p-AKT and p-mTOR expression in HeLa and SiHa cells by MK-2206 and rapamycin inhibited proliferation of cervical cancer cells and indicated that BMX promoted the proliferation of cervical cancer cells through the PI3K/AKT/mTOR pathway.

**Figure 6:**
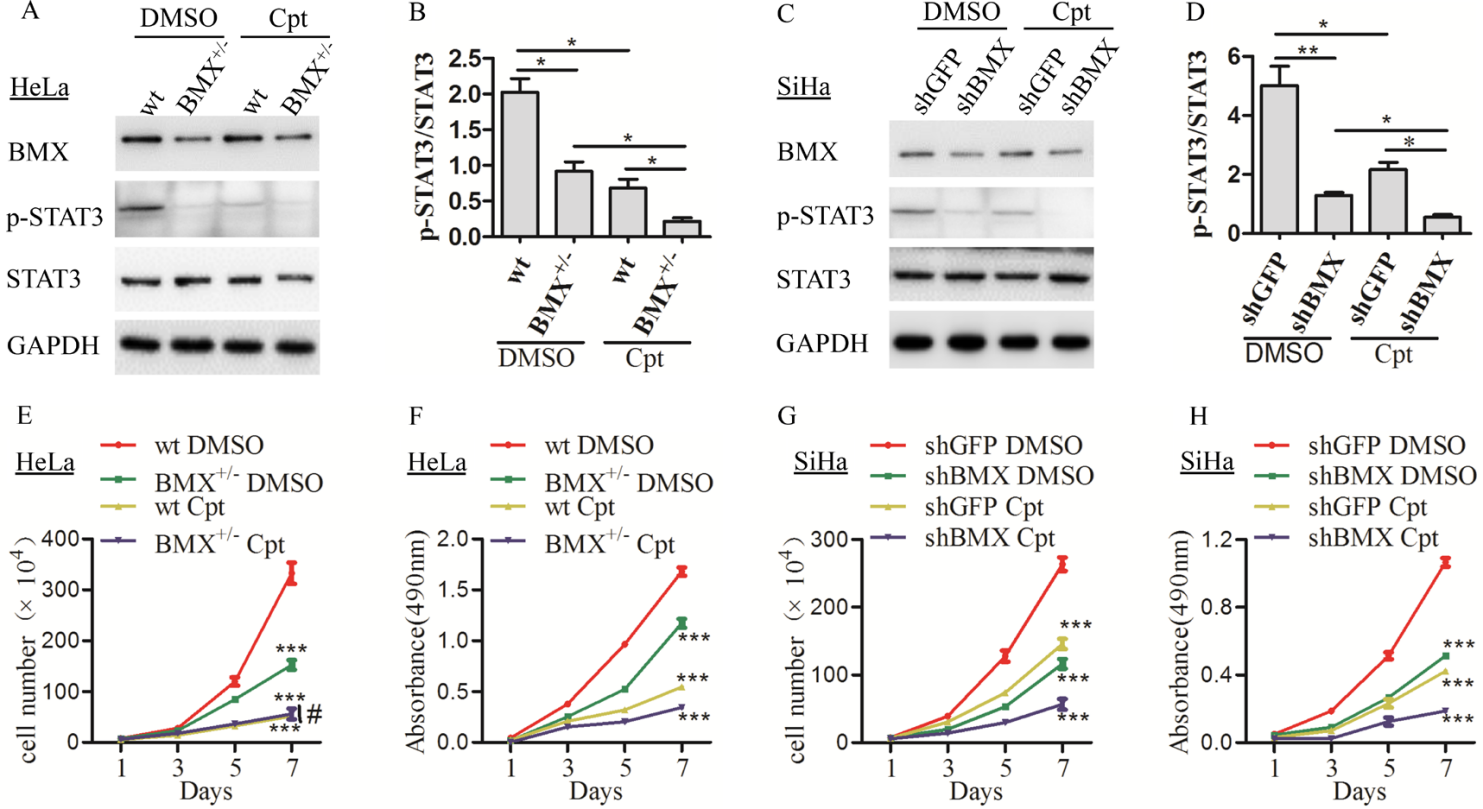
BMX promoted the proliferation of cervical cancer cells through the STAT3 pathway (**A**) HeLa-wt/BMX^+/−^ clones were treated with DMSO control or the STAT3 inhibitor cryptotanshinone (2 μM), and BMX, p-STAT3, and STAT3 expression was detected using western blotting. The quantitative analysis of is shown in (**B**). (**C**) SiHa-shGFP/shBMX clones were treated with DMSO control or the STAT3 inhibitor cryptotanshinone (3 μM), and BMX, p-STAT3, and STAT3 expression was detected using western blotting. The quantitative analysis of is shown in (**D**). (**E**) Growth curves and (**F**) MTT assay results for HeLa-wt DMSO, BMX^+/−^ DMSO, wt cryptotanshinone and BMX^+/−^ cryptotanshinone cells are shown. (**G**) Growth curves and (**H**) MTT assay results for SiHa-shGFP DMSO, shBMX DMSO, shGFP cryptotanshinone and shBMX cryptotanshinone cells are shown. The data were compared as follows: BMX-knockdown (HeLa-BMX^+/−^ and SiHa-shBMX) groups vs. control (HeLa-wt and SiHa-shGFP) groups and all groups treated with cryptotanshinone vs. the corresponding groups treated with DMSO. Western blotting data were determined using a *t*-test. Growth curve and MTT assay data were analyzed using *two-way* ANOVA, and are shown as the mean ± SEM. *p < 0.05, **p < 0.01, ***p < 0.001, ^#^*p* > 0.05.

To confirm that BMX promotes cell proliferation and tumor formation through the STAT3 pathway in cervical cancer cells, the STAT3 inhibitor cryptotanshinone was used to inhibit the expression of p-STAT3 in HeLa-wt, HeLa-BMX^+/−^, SiHa-shGFP and SiHa-shBMX cells. As shown in Figure 6A–6D, the expression of p-STAT3 was much lower in the cryptotanshinone-treated HeLa-wt/BMX^+/−^ and SiHa-shGFP/shBMX cells than in the DMSO-treated cells. In addition, the cryptotanshinone-treated HeLa-wt, HeLa-BMX^+/−^ (Figure 6E and 6F), SiHa-shGFP and SiHa-shBMX (Figure 6G and 6H) cells grew much slower than the DMSO-treated cells, suggesting that suppression of p-STAT3 expression in HeLa and SiHa cells by cryptotanshinone inhibited cell proliferation. These results indicated that BMX can promote the proliferation of cervical cancer cells by enhancing the activity of STAT3. All of these results suggested that BMX promotes the proliferation of cervical cancer cells by activating the PI3K/AKT/mTOR and STAT3 pathways.

## DISCUSSION

**Figure 7:**
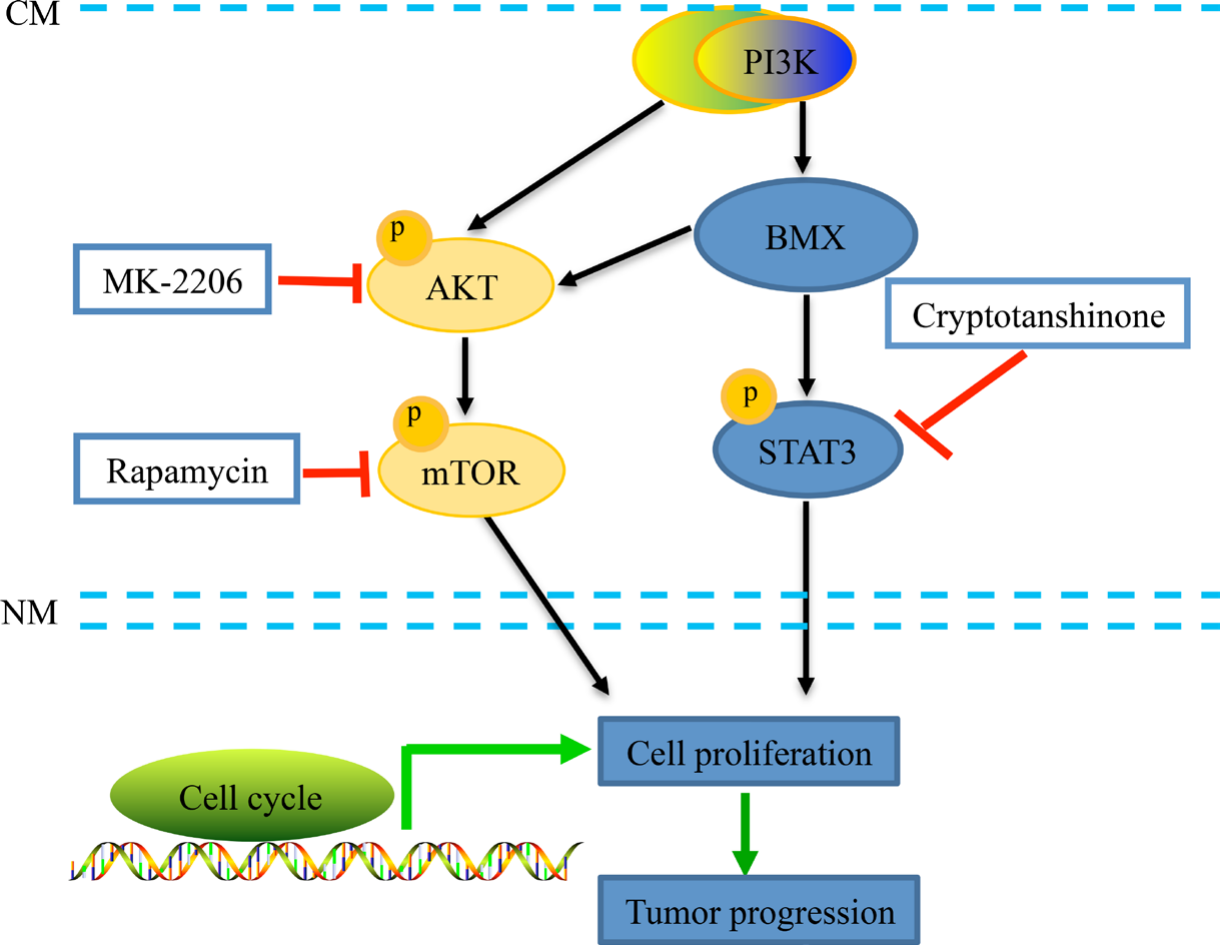
A schematic diagram of the BMX-mediated AKT and STAT3 pathway activation in human cervical cancer cells Upon stimuli, BMX, as well as AKT, was activated by tyrosine phosphorylation of downstream PI3K via binding to PIP3 through the PH domain [16, 33, 34]. BMX could activate the phosphorylation of AKT and STAT3. Using three specific inhibitors of the AKT/mTOR and STAT3 pathways, the allosteric AKT inhibitor MK-2206, mTOR inhibitor rapamycin, and STAT3 inhibitor cryptotanshinone, cell proliferation was further inhibited in BMX-knockdown groups. These findings provide new evidence of the BMX function in cell proliferation and carcinogenesis of cervical cells through the PI3K/AKT/mTOR and STAT3 pathways.

Based on the various literatures, different mechanisms underlying the development of cervical carcinoma were proposed, mainly including phosphatidylinositol-3 kinase (PI3K)/Akt/mTOR [44–46], the mitogen-activated protein kinases (MAPKs) extracellular signal-related kinase (ERK1/2) [47, 48], Janus kinase 2 (JAK2)/signal transducer and activator of transcription-3 (STAT3) [49], JAK3/STAT5 [50], NF-κB [51], Wnt/β-catenin [52], and c-Jun N-terminal kinase (JNK)/p-38 [53] signaling pathways. The whole of data suggests that PI3K/AKT and STAT3 are convergence points of tumorigenic pathways in cervical carcinoma. A previous study reported that overexpression of BMX in bladder cancer cells elevated the activity of AKT and STAT3, whereas knockdown of BMX had the opposite effect [54]. Vogt PK *et al*. reported that an interdependence between PI3K and STAT3, and BMX may be a candidate for mediating the alliance between PI3K and STAT3 [55]. However, the connection between PI3K and BMX and STAT3 has not been explored. In this study, we first reported that BMX can promote cell proliferation and tumor progression through the PI3K/AKT/mTOR and STAT3 pathways. As modeled in Figure 7, these findings provide new evidence of BMX function in cervical carcinogenesis.

BMX is a member of the TEC kinase family, which is the second-largest non-receptor protein tyrosine kinase family. BMX participates in the immune response and inflammation and cytokine signaling in hematopoietic cells, endocardium, and arterial endothelium, as well as in other cells. [56–59]. Moreover, BMX is expressed in several cancers and involved in cell growth, transformation, migration, survival, apoptosis and tumorigenicity [40, 54, 59, 60].

We have verified that BMX promoted the cell proliferation *in vitro* and tumorigenesis *in vivo*. Tumorigenesis study *in vivo* showed that knockdown of BMX significantly diminished tumor growth in immuno-deficient mice (Figure 3). However, the *in vitro* study exhibited the limited effect on growth inhibition (Figure 2H and 2K) and cell cycle arresting (Figure 4E and 4G) when inhibition of BMX through genetic manipulation. We think main reasons of differences between the results from *in vitro* and *in vivo* are following. On one hand, the inhibition effect of BMX is not good. Compared the proliferation (Figure 2C, Supplementary Figure 2A and Figure 2E, Supplementary Figure 2D vs. Supplementary Figure 2C and 2F) and cell cycle (Figure 4A, 4B and Figure 4C, 4D vs. Supplementary Figure 3A, 3B and Supplementary Figure 3C, 3D) results from two inhibitors BMX-IN-1 and LFM-A13, respectively, the differences are more significantly from the data of BMX-IN-1, a potent, selective, and irreversible BMX kinase inhibitor, than LFM-A13, a pharmacologic reversible inhibitor of BMX. Choosing the BMX^−/−^ clones is vital, but it's a pity that we didn't obtain BMX^−/−^ clones in cells. On the other hand, besides cell cycle changes, apoptosis also worked between the knockdown of BMX and the control cells (Supplementary Figure 4). The percentage of apoptosis cells was higher in the inhibition of BMX through pharmacological and genetic manipulation cells than in the corresponding control cells.

Furthermore, an AKT inhibitor (MK-2206), mTOR inhibitor (rapamycin) and STAT3 inhibitor (cryptotanshinone) were used in HeLa-wt, HeLa-BMX^+/−^, SiHa-shGFP and shBMX cell lines. In BMX-knocked down or silenced Hela or SiHa cells, the residual p-AKT, p-mTOR and p-STAT3 expression might be abolished by the AKT, mTOR and STAT3 specific inhibitors, respectively, thus affected further growth and survival. It could be possible that BMX cooperates with AKT/mTOR and STAT3 to support the cell proliferation and tumorigenesis. BMX alone is not sufficient to lead normal cervical C-33A cells towards a tumorigenic phenotype. This can support the idea that BMX needs to cooperate with other pathways *in vivo* tumorigenesis of cervical cancer cells. In conclusion, our studies have found BMX can promote cell proliferation and tumorigenesis in cervical cancer cells, thus the development of BMX inhibitors, like BMX-IN-1, is necessary for cervical cancer therapy.

## MATERIALS AND METHODS

### Cell lines and clinical samples

The human cervical carcinoma cell lines HeLa, SiHa, C-33A, HT-3 and CaSki were purchased from the American Type Culture Collection (ATCC, USA). HeLa, SiHa and C-33A cells were cultured in Dulbecco's Modified Eagle's Medium-high glucose (DMEM; Sigma-Aldrich, St Louis, Mo) supplemented with 10% fetal bovine serum (FBS; Invitrogen, Carlsbad, Calif). HT-3 and CaSki cells were cultured in McCoy's 5A and RPMI-1640 (Sigma-Aldrich), respectively, supplemented with 10% FBS. All cell lines were incubated at 37°C in an atmosphere containing 5% CO_2_.

Clinical samples including 43 normal cervix (NC), 25 cervical carcinoma *in situ* (CIS) and 52 invasive cervical carcinoma (ICC) samples were obtained from the First Affiliated Hospital of Xi'an Jiaotong University between 2005 to 2011. This study was approved by the Ethics Committee for the Medical College of Xi'an Jiaotong University. None of the patients had received chemotherapy, immunotherapy or radiotherapy, and all of them provided their informed consent before sample collection.

### Immunohistochemistry and immunocytochemistry

Formalin-fixed, paraffin-embedded tissue samples were sliced into 4-μm sections and placed in a 60°C incubator for 3–12 h. After deparaffinization and rehydration, antigen retrieval was performed with citrate buffer in a steam pressure cooker for 2 min, and sections were cooled quickly to room temperature. Endogenous peroxidase was blocked with 3% H_2_O_2_, and washed with PBS. The sections were incubated at 4°C overnight with the following primary antibodies: anti-BMX (1:150, 610793, BD, USA) and anti-Ki67 (1:100, sc-23900, Santa Cruz Biotechnology). They were then incubated with secondary antibodies (Vector Laboratories, Burlingham, CA) at room temperature for 20 min, visualized with 0.05% DAB (3,3′-diaminobenzidine) and counterstained with hematoxylin. The primary antibody was replaced with PBS for the negative control.

Immunocytochemistry was performed as described above. Cells were cultured on coverslips, fixed in 4% paraformaldehyde for 30 min at room temperature, permeabilized with 0.2% Triton X-100 for 20 min, and then blocked and incubated as described above.

All slides were examined using an Olympus-CX31 microscope (Olympus, Tokyo, Japan) and scored by two investigators that analyzed five randomly selected fields at ×40 or ×100 magnification. BMX staining was represented using an IRS that was determined by multiplying the values for staining intensity (scored as 0, no staining; 1, weak staining; 2, moderate staining; or 3, strong staining) and the values for the percentage of positive cells (scored as 0, < 10%; 1, 10%–25%; 2, 25–50%; 3, 50–75%; or 4, 75–100%) in each sample. BMX staining was also classified into two categories, in which an IRS of >3 was defined as positive and the others as negative.

### Western blot analysis

Cells and clinical tissues were lysed for 30–45 mins on ice in lysis buffer containing freshly added protease inhibitor cocktail (Roche Diagnostics, USA). After BCA quantification (Pierce, USA), the protein was added to 5× loading buffer and boiled at 95°C for 10 min. Equal amounts of protein were separated by SDS-PAGE and blotted onto activated polyvinylidene difluoride membranes (Millipore, USA). After blocking, the membranes were incubated with primary antibodies overnight at 4°C. The antibodies used were as follows: BMX (1:2000, 610793, BD), phospho-Etk (Tyr40) (1:500, #3211, Cell Signaling Technology), AKT1 (1:500, sc-5298, Santa Cruz Biotechnology), p-AKT (1:1000, #4060, Cell Signaling Technology), mTOR (1:1000, A2445, ABclonal), p-mTOR (1:1000, AP0094, ABclonal), STAT3 (1:1000, #9132, Cell Signaling Technology), phospho-STAT3 (Tyr705) (1:1000, #4113, Cell Signaling Technology), β-actin and GAPDH (1:1000, Santa Cruz Biotechnology). Blots were incubated with secondary antibodies coupled to horseradish peroxidase (Thermo Fisher Scientific, USA) for 1 h and visualized using ECL detection (Millipore) on X-ray film. Relative quantitation was analyzed with Quantity One software.

### Vectors construction

For BMX-TALEN construction, software (https://tale-nt.cac.cornell.edu/) was used to design the TALEN genomic binding site left arm (5′-ggatacaaaatctattct-3′) and right arm (5′-CTTTTGCTGTGATCTTTT-3′), and the target sequence between the two binding sites was 16 bp in length (Supplementary Figure 1A). Different TAL repeat modules were added in accordance with the order and linked into the TALEN vectors (L15 and R11) using a PCR-based protocol according to instructions provided by SiDanSai Biotechnology Co., Ltd (Shanghai, China). The DNA sequences of IRES, AcGFP and DsRed were amplified using PCR primers: IRESFXhoI (5′-ATTCTCGAGTTATCCGCCCCTCTCCCTC-3′), IRE -SRXbaI (5′-GCTCTAGATGTGGCCATATTATCA TCG-3′), AcGFPFXbaI (5′-GCTCTAGAACCATGGTGA GCAAGGGCG-3′), AcGFPRApaI (5′-GTTGGGCCCG CTCACTTGTACAGCTCAT-3′), DsRedFXbaI (5′-GCTC TAGAACCATGGCCTCCTCCGAGA-3′) and DsRed RApaI (5′-GTTGGGCCCGCTACAGGAACAGGTGGT-3′) and then ligated into the preliminary BMX-TALEN plasmids to generate two new plasmids (BMX-TALEN-L15-IREA-AcGFP and BMX-TALEN-R11-IREA-DsRED, Supplementary Figure 1B). All constructs were verified by sequencing.

The BMX-specific short hairpin RNA (shBMX) and control shRNA GFP was purchased from GenePharma Co., Ltd. (Shanghai, China).

For the BMX-overexpression plasmid, full-length BMX cDNA was amplified from the plasmid pcDNA3.1-BMX wt (a kind gift from Professor Chi-Ying F. Huang). The primer sequences were designed as follows: BMXF (5′-3′)- CGCGTCGACATGGATACAAAATCTATTCTA; and BMXR (5′-3′)-CTAGGTACCTCAATGCTTGTCT TTTTCCCG. The product was cleaved with *SalI* and *KpnI* and ligated into the vector pCAG-IRES2-AcGFP1 (Clontech, Mountain View, Calif) to generate the pCAG-IRES2-AcGFP-BMX recombinant plasmid, which was then verified by sequencing.

### Transfection and sorting

After transfection of HeLa cells with the BMX-TALEN vectors (left:right = 1:1, 2 μg of each plasmid) using Lipofectamine 2000 reagent for 24–48 h, the cells were classified using fluorescence-activated cell sorting (FACS, BD Biosciences) based on the expression of the AcGFP and DsRed fluorescent markers. The AcGFP^+^/DsRED^+^ cells were plated on 10-cm culture plates at a low density in growth medium for approximately 10 days. Then, individual colonies were picked and cultured. When grown to 70%–90% confluency, the cells were collected and lysed to extract protein and then the loss of BMX expression was confirmed using a western blotting assay to discriminate clones. We then identified candidate clones by sequencing. Briefly, genotyping at the TALEN target site was amplified for each colony using PCR (98°C, 1 s; 55°C, 5s; 72°C, 15s) using a Thermo Scientific Phusion Human Specimen Direct PCR kit (Thermo Scientific) and a primer pair (F5′-TTTGATAAGGTGGTCTGGA-3′; R5′-AGAGGATCTTCACAGTGTA-3′) designed to yield a 564 bp amplicon around the target site. Amplicons were subcloned using a TA Cloning Kit for sequencing (Life Technologies). In comparison with the other wild-type sequence, two BMX^+/−^ mutants were characterized, which contained 2 bp and 28 bp deletions and harbored a frameshift mutation (Supplementary Figure 1C).

The shRNA and BMX-overexpression vector were transfected into SiHa and C-33A cells, respectively, using Lipofectamine 2000 reagent (Invitrogen, Carlsbad, CA, USA) according to the product description. The transfected cells were selected using G418 (Calbiochem, La Jolla, CA, USA, SiHa 1000 μg/mL; C-33A 500 μg/mL) for two weeks, and then, single colonies were picked, cultured and identified using western blotting.

### Cell proliferation and viability assays

1–4 × 10^4^ cells were plated at the appropriate density in 35-mm culture dishes with 2 mL of medium. Cells were collected, and then counted on days 1, 3, 5, and 7 using a hemocytometer. Cell growth curves were generated to measure cell proliferation.

Cell viability was measured using a 3-(4,5- dimethylthiazol-yl)-2,5-diphenyltetrazolium bromide (MTT, Sigma-Aldrich) assay. Cells were plated at a density of 1–2 × 10^3^ cells in 96-well plates with 200 μL of medium. Following the standard protocol, the plates were assessed on days 1, 3, 5, and 7 by measuring the absorbance at 490 nm (Bio-Rad).

Cells were seeded in complete medium containing DMSO or inhibitors (BMX-IN-1, MCE, USA; LFM-A13, Millipore and MCE, USA; MK-2206, rapamycin, cryptotanshinone, Selleck, USA) at an appropriate dose, and cell viability was measured using an MTT assay. In addition, the medium containing LFM-A13 was changed every 24 h.

### Flow cytometry analysis

Cells (1 × 10^6^) were seeded in 60-mm culture dishes for 24–48 h and labeled with 30 μM Brdu for 30–60 mins. The cells were harvested, fixed, penetrated, denatured and stained with anti-APC-Brdu using APC-Brdu cell proliferation Detection Kit (KeyGEN BioTECH), then analyzed using a FACS Calibur flow cytometer (BD Biosciences, USA).

Cells were harvested when grown to 50%–70% confluency and fixed in 70% cold ethanol overnight at 4°C. After being washed twice with PBS, all samples were incubated in RNase A and propidium iodide (Sigma-Aldrich) for 30 min in the dark and then analyzed using a FACS Calibur flow cytometer (BD Biosciences, USA). Cell cycle distribution was analyzed using FlowJo 7.6 software.

Apoptosis was analyzed using Annexin V-PE/7-AAD Apoptosis Detection Kit (BD Biosciences, USA) according to the manufacturer's instructions.

### Tumor xenograft experiment

The animal experiments were approved by the Animal Care and Use Committee of the Medical School of Xi'an Jiaotong University. Female BALB/c nude mice (4 to 6 weeks old) were purchased from Slac Laboratory Animal Co., Ltd. (Shanghai, China) and fed in the Medical College Experimental Animal Center of Xi'an Jiaotong University. Cells (1 × 10^6^) mixed with Matrigel (BD, USA) in 200 μL total volume were injected into subcutaneous tissue (6 mice per group). The tumor size was measured weekly, and the volume was calculated using the following formula: Volume = (length × width^2^)/2. Last, mice were killed by cervical dislocation, and tumors were dissected, weighed, fixed with 4% paraformaldehyde solution and paraffin-embedded for immunohistochemical analysis.

### Statistical analysis

Statistical analyses were performed using GraphPad Prism V5.01 software (La Jolla, USA). Sample response rate was analyzed with a chi-square test. Univariate analysis was performed using Student's *t*-test (two groups) or one-way ANOVA (three or more groups). Two-factor analysis of variance was analyzed using two-way ANOVA. In all tests, a value of *p* < 0.05 was defined as statistically significant, and the data are shown as the mean±SEM.

## SUPPLEMENTARY FIGURES


