# Supplementary material for: BMX/Etk promotes cell proliferation and tumorigenicity of cervical cancer cells through PI3K/AKT/mTOR and STAT3 pathways
Source: Oncotarget. 2017 Apr 27;8(30):49238–52. doi: 10.18632/oncotarget.17493 (PMC5564764; doi:10.18632/oncotarget.17493)
Supplement: Supplementary file 1 [file oncotarget-08-49238-s001.pdf]

BMX/Etk promotes cell proliferation and tumorigenicity of cervical cancer cells through PI3K/AKT/mTOR and STAT3 pathways

Supplementary Materials

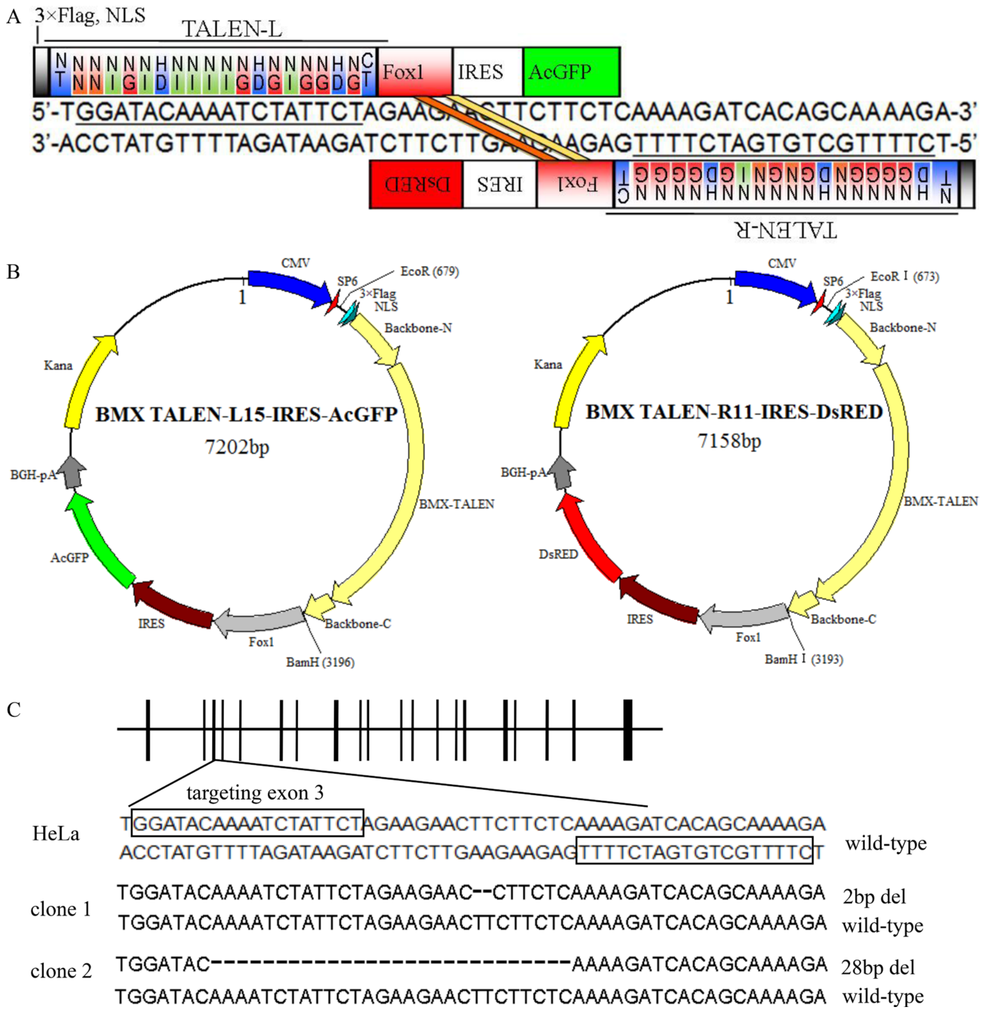

Supplementary Figure 1: Efficient and rapid BMX-expressing changed clones were successfully achieved. (**A**) Schematic overview depicting the strategy for targeting the BMX gene using TALENs, which is annotated with the following codes: (NI = A, HD = C, NN = G, NG = T) [61]. AcGFP, green fluorescent protein; DsRed, red fluorescent protein; NLS, nuclear localization signal; TALEN-L, TALE repeat monomers that binds to the left target sequence; TALEN-R, TALE repeat monomers that bind to the right target sequence. (**B**) The BMX-TALEN plasmids (BMX-TALEN-L15-IREA-AcGFP and BMX-TALEN-R11-IREA-DsRED) are shown. (**C**) Generation of BMX frameshift mutant clones with TALENs targeting exon 3 in HeLa cells. Boxes indicate the TALEN targeting sequence. The 2 bp and 28 bp deletions in one allele of the clones are indicated.

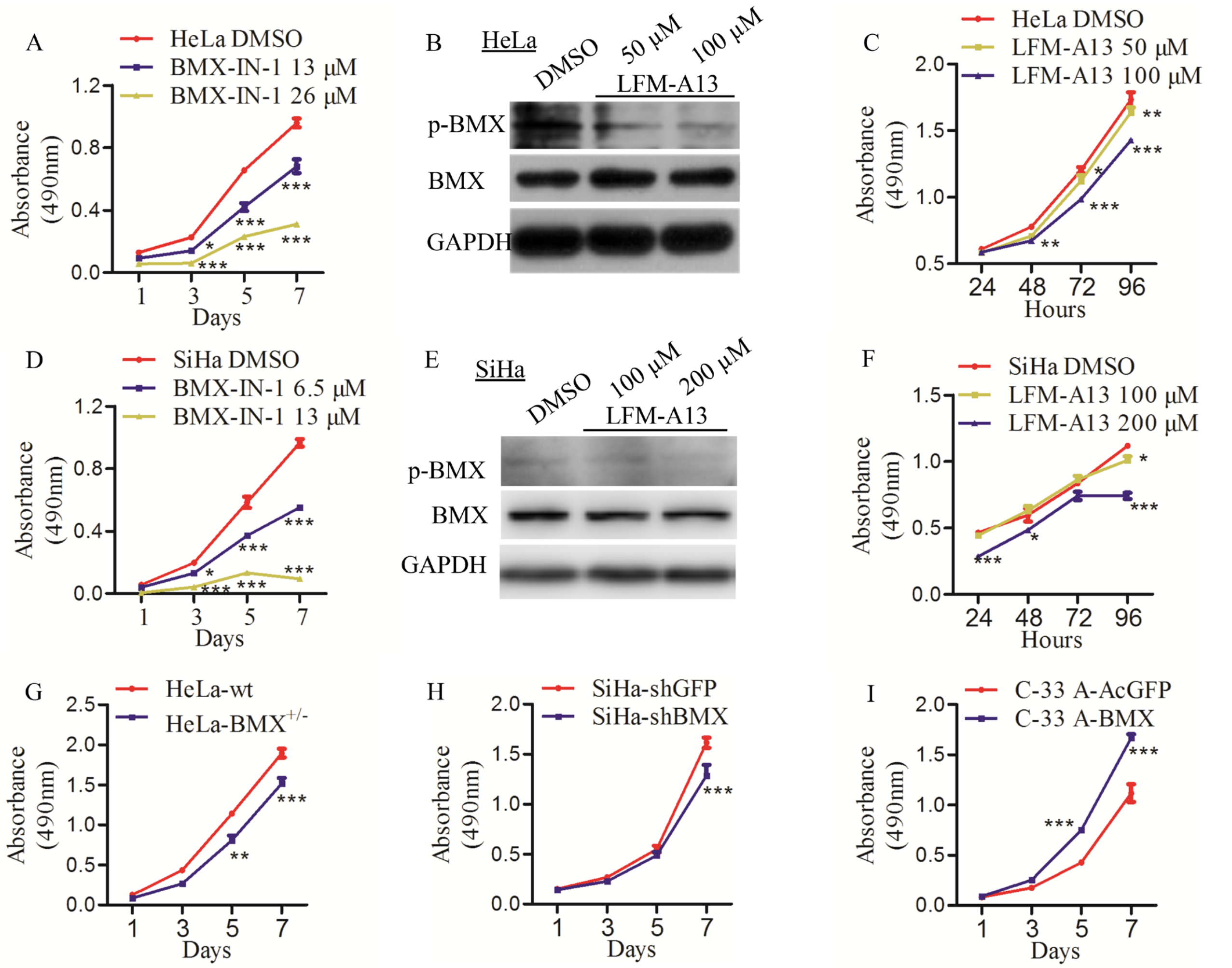

Supplementary Figure 2: Cell viability was detected by MTT in BMX-expression changed cells *in vitro*. (**A**) HeLa and (**D**) SiHa cells were treated with DMSO, and BMX-IN-1 at an appropriate dose, MTT was used to assess the viability at day 1, 3, 5 and 7. (**B**) HeLa and (**E**) SiHa cells were treated with DMSO and LFM-A13, a pharmacologic inhibitor of BMX, and the phosphorylation and total BMX levels were determined using a western blot analysis. (**C**) HeLa and (**F**) SiHa cells were treated with DMSO and LFM-A13, MTT was used to assess the viability at the 24 h, 48 h, 72 h and 96 h time points. The viability of (**G**) HeLa-wt/BMX$^{+/-}$, (**H**) SiHa-shGFP/shBMX and (**I**) C-33A-AcGFP/C-33A-BMX cells was detected by MTT assay. Values are shown as the mean±SEM from three independent experiments (*t*-test, *$p < 0.05$, **$p < 0.01$, ***$p < 0.001$ vs the corresponding control).

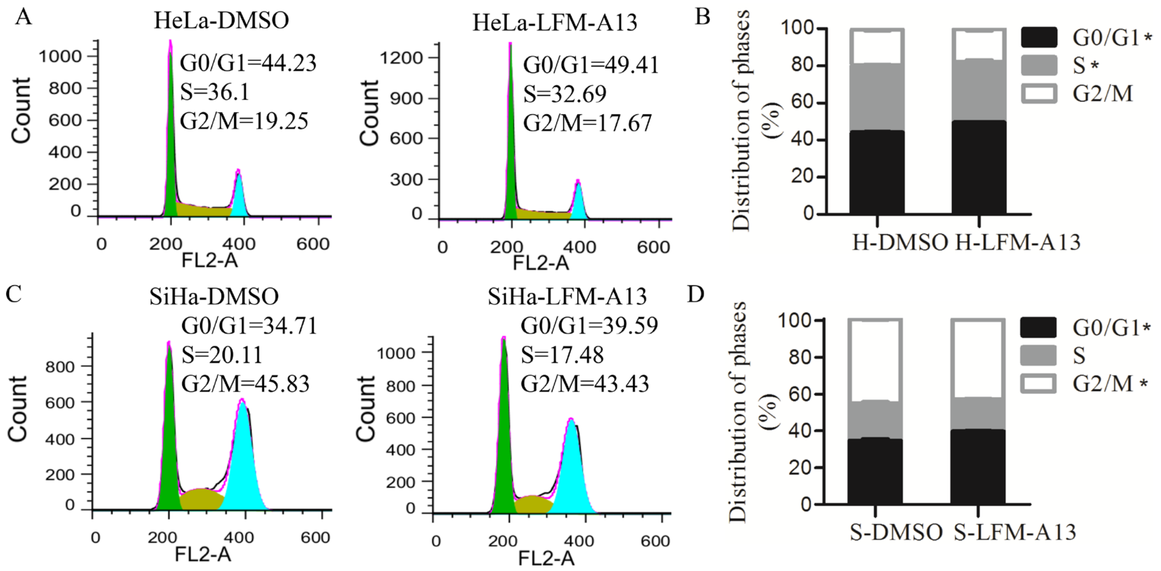

Supplementary Figure 3: Cell cycle distribution after treated with LFM-A13. (**A**) Pretreated HeLa cells with DMSO and 50 μM LFM-A13, the cell cycle distribution was analyzed using flow cytometry analysis, and the statistical analysis is shown in (**B**). (**C**) Pretreated SiHa cells with DMSO and 200 μM LFM-A13, the cell cycle distribution was analyzed, and the statistical analysis is shown in (**D**). A *t*-test was used for the statistical analysis, *$p < 0.05$, **$p < 0.01$.

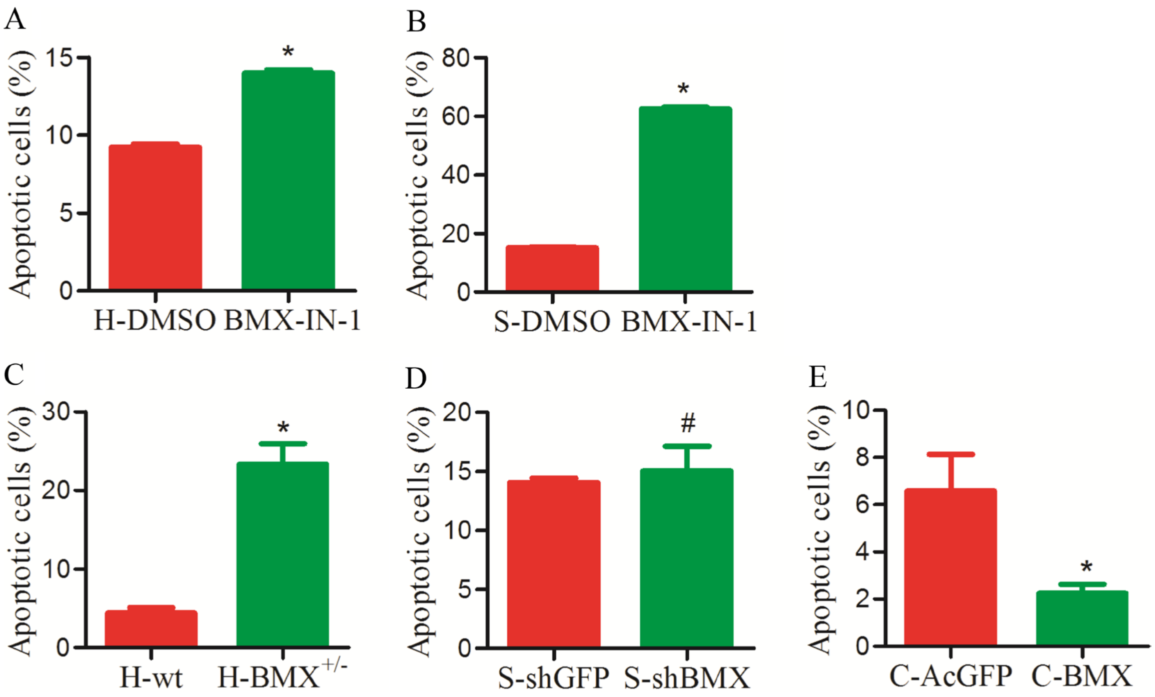

Supplementary Figure 4: Apoptotic cells analyzed when the expression changed of BMX through pharmacological and genetic manipulation. Treated HeLa cells with DMSO and 26 μM BMX-IN-1 (**A**), SiHa cells with DMSO and 13 μM BMX-IN-1 (**B**), respectively, the apoptotic cells were detected using flow cytometry analysis, and the statistical analysis was shown. The apoptotic cells of HeLa-wt/HeLa-BMX$^{+/-}$ (**C**), SiHa-shGFP/SiHa-shBMX (**D**), and C-33A-AcGFP/C-33A-BMX (**E**) cells were analyzed. A *t*-test was used for the statistical analysis, $^{*}p < 0.05$, $^{\#}p > 0.05$.

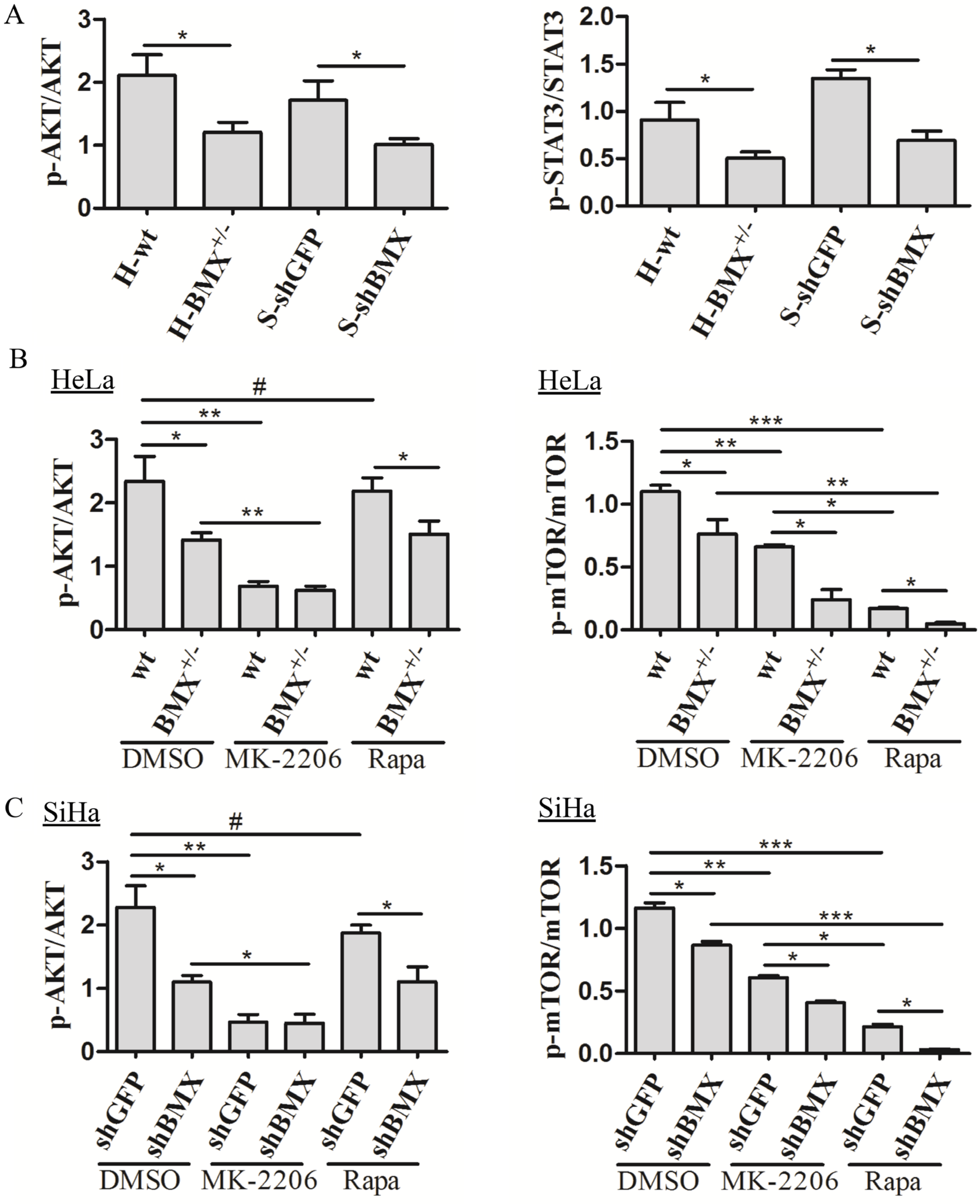

Supplementary Figure 5: BMX activated the phosphorylation of AKT/mTOR and STAT3. (**A**) The relative quantitative analysis of Figure 5A. Comparisons of p-AKT/AKT (Left) and p-STAT3/STAT3 (Right) was shown in HeLa-wt/HeLa-BMX$^{+/-}$ and SiHa-shGFP cells. (**B**) The relative quantitative analysis of Figure 5B. Comparisons of p-AKT/AKT (Left) and p-mTOR/mTOR (Right) was shown in HeLa-wt/HeLa-BMX$^{+/-}$ cells treated with DMSO, the selective AKT inhibitor MK-2206 and mTOR inhibitor rapamycin. (**C**) The relative quantitative analysis of Figure 5C. Comparisons of p-AKT/AKT (Left) and p-mTOR/mTOR (Right) was shown in SiHa-shGFP/SiHa-shBMX cells treated with DMSO, the selective AKT inhibitor MK-2206 and mTOR inhibitor rapamycin. The data were determined using a t-test, and are shown as the mean ± SEM. *$p < 0.05$, **$p < 0.01$, ***$p < 0.001$, $^{\#}p > 0.05$.
